# AEP promotes aberrant RNA splicing through DDX3X cleavage in solid tumors

**DOI:** 10.1172/JCI177609

**Published:** 2024-02-01

**Authors:** Yadong Xie, Haohao Zhang, Xinyang Song

**Affiliations:** State Key Laboratory of Cell Biology, Center for Excellence in Molecular Cell Science, Shanghai Institute of Biochemistry and Cell Biology, Chinese Academy of Sciences, University of Chinese Academy of Sciences, Shanghai, China.

## Abstract

Aberrant alternative splicing (AS) events have been identified in a variety of cancers. Although somatic mutations of splicing factors and dysregulation of RNA-binding proteins (RBPs) have been linked to AS and tumor malignancy, it remains unclear how upstream mechanisms contribute to cancer development via alternative gene splicing. In this issue of the *JCI*, Wenrui Zhang and colleagues identified the role of asparagine endopeptidase (AEP), an intracellular cysteine endopeptidase, in promoting solid tumor–associated RNA splicing. The authors demonstrated that tumor environmental factors such as oxygen and nutrient deprivation induce the activity of AEP in a HIF1A-dependent manner. The activated AEP, in turn, cleaves an RNA helicase DDX3X to promote its nuclear retention. The authors further showed that this DDX3X nuclear fraction engages with splicing machinery to induce AS events in several cancer cells. These findings suggest that targeting an AEP-dependent aberrant RNA splicing cascade may facilitate therapeutics for solid tumors.

## Aberrant RNA splicing in cancers

Alternative splicing (AS) is a critical cellular process that contributes to the encoding capacity of transcriptomes of eukaryotic cells (1). Recently, high-throughput sequencing and genome-wide analyses have uncovered dysregulated AS events in many types of cancers (2, 3). These cancer-associated AS events lead to the biogenesis of aberrant RNA isoforms. In turn, their encoded protein products promote the growth and survival of tumor cells and eventually contribute to tumor malignancy (2, 3).

RNA splicing is a finely regulated enzymatic process that requires the coordination of the spliceosome machinery, cis-acting elements, and trans-acting factors (1). Genetic mutations of RNA splicing components or oncogene-relevant transcriptional changes of these components alter the functions of RNA splicing machinery in cancer cells and generate cancer-specific AS events (2, 3). For example, frequent mutations in genes encoding regulatory splicing factors or even the core spliceosomal proteins (SRSF2, U2AF1, and SF3B1) and their impacts on AS have been reported in either acute or chronic leukemia (4–6). In addition, the activation of oncogenic MYC is required for dysregulation of spliceosomal genes and tumor malignancy (7, 8). One recent study analyzed the sequencing datasets of 11 cancer types from The Cancer Genome Atlas (TCGA) program and revealed that some AS changes affect protein domains that are also mutated in cancers (9). However, the number of AS changes were negatively correlated with the number of somatic mutations in driver genes, indicating other uncharacterized mechanisms may also contribute to the tumor AS biogenesis (9).

## AEP promotes tumor AS events via DDX3X cleavage

In this issue of the *JCI*, Wenrui Zhang and colleagues show that within a hostile tumor microenvironment asparagine endopeptidase (AEP) triggers an AS-biogenesis cascade in solid tumors by cleaving the RNA helicase DDX3X (10). AEP, also called legumain (LGMN), is a member of the C13 family of cysteine proteases that cleave protein substrates after asparagine residues (11). Despite its important role in maintaining tissue homeostasis, AEP is highly expressed and has been indicated as a biomarker of poor prognosis in several types of solid tumors, especially in glioblastoma and breast cancer (12). Dr. Lin leads a team that has long been engaged in studying the functional mechanisms of AEP in solid tumors. Their previous works revealed the tumor suppressor P53 and an actin regulator TMOD3 as AEP substrates, suggesting protease-mediated protein cleavage can generate altered products with tumor exacerbation roles (13, 14).

In the present study, the authors identified the DEAD-box helicase family member DDX3X as a substrate of AEP by mass spectrometry-based immunoprecipitation proteomics. Intriguingly, DDX3X is an RNA-binding protein (RBP) that is associated with a poor prognosis as AEP in both glioblastoma and breast cancer patients. By endogenous coimmunoprecipitation, the investigators validated the interaction between AEP and DDX3X in several glioblastoma and breast cancer cell lines under hypoxia and nutrient deprivation, and they also revealed that DDX3X binds to AEP via its N-terminal region (10).

Since hypoxia and starvation activate HIF1A signaling leading to the maturation of AEP, the binding of DDX3X to AEP resulted in a cleavage event at its Asn124 site and generated two truncated proteins, referred to as tDDX3X-N (amino acid 1-124) and the carboxyl-terminal DDX3X (tDDX3X-C) (amino acid 125-662) in tumor cells. The full-length DDX3X is located in both the cytoplasm and nucleus of tumor cells. However, after the cleavage by AEP, an N-terminal nuclear export signal (NES) was removed from DDX3X, causing a nuclear retention of its truncated protein (namely tDDX3X-C in Zhang et al.). As AEP was positively correlated with tumor malignancy, silencing of AEP reduced tumor progression while overexpression of tDDX3X-C in AEP-silenced tumor cells restored their aberrant proliferation property, indicating tDDX3X-C is an AEP downstream effector that contributes to tumor progression (10).

**Figure 1:**
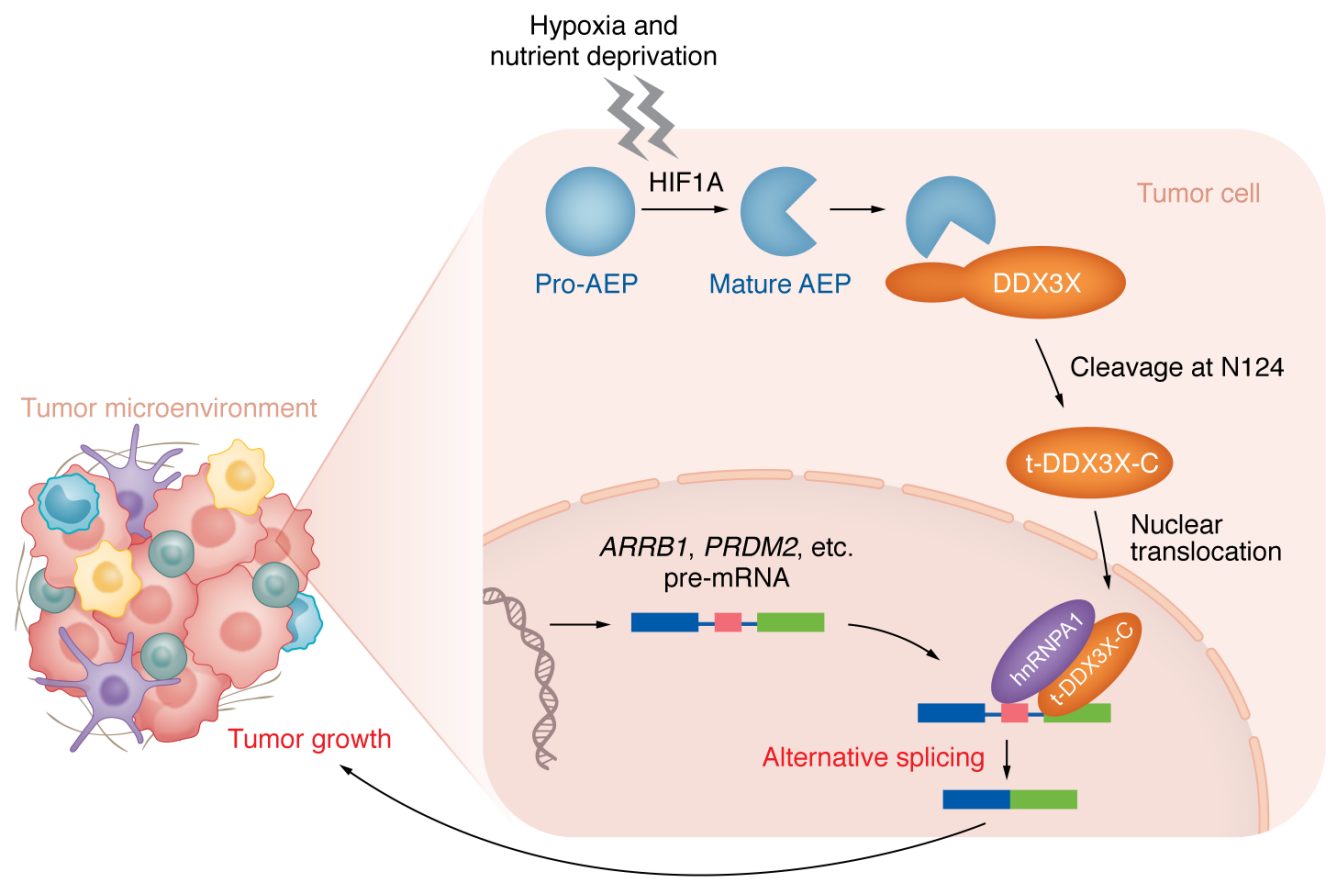
AEP cleaves DDX3X to drive tumor-promoting AS events. Tumor microenvironmental stresses, such as hypoxia and nutrient deprivation, activate AEP from its inactive form (pro-AEP) into the mature form through HIF1A. AEP cleaves DDX3X at amino acid 124 to induce its nuclear translocation. Once in the nucleus, t-DDX3X-C affects downstream tumor AS events in a hnRNPA1-dependent manner. Examples of pre-mRNAs affected include transcripts that encode ARRB1, involved in the regulation of glycolysis, and PRDM2, which is important for tumor suppression.

To understand how tDDX3X-C affects tumor progression, Zhang and investigators examined the transcriptome of tDDX3X-C–overexpressed tumor cells (10). In particular, they used rMATS software to profile the RNA splicing events in these cells (15), and several types of AS events were observed in their study. Mechanistically, Zhang et al. showed that tDDX3X-C can bind to splicing factor hnRNPA1 to promote tumor-associated AS, while another splicing factor SRSF1 was not involved. To further illustrate how AEP/ tDDX3X-C-induced AS contributes to tumor growth, they verified the presence of spliced isoforms for several cancer-related genes including β-arrestin 1 (ARRB1), PR/SET domain2 (PRDM2), and nuclear receptor corepressor 2 (NCOR2). Of note, in comparison to full-length ARRB1, an ARRB1 splicing variant (*Arrb1* without exon 13) strongly promoted tumor growth, likely through regulating tumor glycolysis (Figure 1) (10).

## Conclusions and implications

In this study, Zhang, et al. demonstrate that upstream signaling is involved in triggering tumor-promoting AS events, especially in glioblastoma and breast cancer. The authors conclude that tumor environmental factors such as hypoxia and nutrient deprivation activate HIF1A signaling for the maturation of AEP, which, in turn, cleaves DDX3X to promote its sequestration in the nucleus, thus initiating a nuclear DDX3X/hnRNPA1–dependent AS program in tumor cells. Their findings shed light on the upstream mechanisms that drive dysregulated RNA splicing in tumors and illustrate the potential tumor-promoting roles of the resulting encoded variants (10). The results serve to redirect tumor research toward upstream signaling of AS as a targetable mechanism underlying tumor growth. However, the authors only tested the altered functions of a few tumor suppressor gene variants in in vitro tumor progression assays. The in vivo tumor-supporting roles of these AS variants and whether other AS candidates are involved in tumorigenesis remain to be investigated.

Cancer-associated RNA splicing is linked to dysregulation of RBP expression or somatic mutations in spliceosomal proteins. The emergence of this work suggests that, in alignment with previously described mechanisms, posttranscriptional modification, e.g., proteolytic cleavage, also contributes to RNA missplicing and tumor growth. Zhang et al. highlight the protease AEP as being involved in the cleavage of the RBP factor DDX3X and subsequent AS in glioblastoma and breast cancer (10). Since AEP has been reported to be widely expressed in various solid tumors (12), it would be interesting to determine whether such proteolytic cleavage-triggered AS is a widespread mechanism underlying solid tumor development. In addition, it would be intriguing to explore whether the downstream AS program yields tumor-specific isoforms in different cancer types and whether this posttranscriptional mechanism coordinates with, or is in parallel to, spliceosomal- or other genetic mutation-related AS in promoting tumor progression. Finally, this work may inspire future studies to identify more upstream modulators for RBPs and determine their roles in AS-relevant tumor functions.

In recent years, therapeutic approaches targeting RBP factors have been tested in clinical trials for cancer treatment, indicating the interference of aberrant RNA-protein networks as a promising direction for drug discovery (16). Aligning with the current approaches, the work presented by Zhang, et al. (10) may create an avenue for cancer therapy by targeting the posttranscriptional regulators of RBPs, for example, the cysteine protease AEP.

## Version 1 02/01/2024

Electronic publication
